# Supplementary material for: Impact of COVID-19Quarantine on Low Back Pain Intensity, Prevalence, and Associated Risk Factors among Adult Citizens Residing in Riyadh (Saudi Arabia): A Cross-Sectional Study
Source: Int J Environ Res Public Health. 2020 Oct 6;17(19):7302. doi: 10.3390/ijerph17197302 (PMC7579188; doi:10.3390/ijerph17197302)
Supplement: Supplementary file 1 [file ijerph-17-07302-s001.pdf]

Questionnaire S1: COVID-19 and Back Pain

This questionnaire is being conducted in order to obtain information about your potential back pain. The main purpose is to investigate back pain occurrence during quarantine measures caused by the COVID-19 outbreak. It is entirely anonymous and data obtained will be used only for scientific purposes. Please fill the questionnaire with maximal accuracy and honesty.

Thank you for your cooperation

Fill or <u>underline</u>

* Required questions

1. Personal data:

Gender*	Age *	Height in cm *	4. Weight in kg *
Female	____________	____________	____________
Male			

2. City where you stayed before the COVID-19 outbreak: *

____

3. City where you stayed during the COVID-19 outbreak: *

____

4. Your occupation before the COVID-19 outbreak: *

Employee

Student

Retired

Other: ________________________

5. Your occupation during the COVID-19 outbreak: *

Employee

Student

Retired

Other: ________________________

6. I suffer from chronic disease/chronic condition: *

Yes

No

If the previous answer is YES, name the diseases/conditions:

____

7. At my job/occupation, BEFORE the COVID-19 quarantine: *

5. I was always sitting

4. I sit most of the time

3. I sit and moved equally

2. I moved around most of the time

1. I moved around always

8. At my job/occupation, DURING the COVID-19 quarantine: *

5. I was always sitting

4. I sit most of the time

3. I sit and move equally

2. I move around most of the time

1. I move around always

9. I worked/studied online BEFORE the quarantine:

Yes

No

10. I started a "home office" or online learning AFTER the COVID-19 outbreak: *

Yes

No

11. BEFORE the COVID-19 quarantine, the back pain was located in .... (multiple choice if needed): *

Nowhere

Neck

Shoulder(s)

Thoracal area

Lower back

Leg(s)

Other: ________________________

12. DURING the COVID-19 quarantine, the back pain is/was located in .... (multiple choice if needed): *

Nowhere

Neck

Shoulder/s

Thoracal area

Lower back

Leg/s

Other: ________________________

13. My back pain BEFORE the COVID-19 quarantine measures was: *

No pain 1 2 3 4 5 Extreme pain

14. My back pain DURING the COVID-19 quarantine measures is: *

No pain 1 2 3 4 5 Extreme pain

15. My physical activity BEFORE the COVID-19 outbreak *

5. 6–7 times a week

4. 4–5 times a week

3. 2–3 times a week

2. Once a week

1. None

16. My physical activity DURING the COVID-19 quarantine: *

5. 6–7 times a week

4. 4–5 times a week

3. 2–3 times a week

2. Once a week

1. None

17. I was following the ergonomic recommendations (e.g., correct posture, walking, sitting) before the COVID-19 quarantine: *

5. Strongly agree

4. Agree

3. Neutral

2. Disagree

1. Strongly disagree

18. I followed the ergonomic recommendations (e.g., correct posture, walking, sitting)

during the COVID-19 quarantine: *

5. Strongly agree

4. Agree

3. Neutral

2. Disagree

1. Strongly disagree

19. My stress level before the COVID-19 outbreak: *

No stress 1 2 3 4 5 Maximal stress

20. My stress level during the COVID-19 outbreak: *

No stress 1 2 3 4 5 Maximal stress

I understand that the questionnaire is anonymous and data obtained will be used only for a scientific purpose. *

☐ I understand

Thank you very much for your time.
